# Melanoma. Shall we move away from the sun and focus more on embryogenesis, body weight and longevity?^☆^

**DOI:** 10.1016/j.mehy.2013.05.031

**Published:** 2013-11

**Authors:** Veronique Bataille

**Affiliations:** Twin Research and Genetic Epidemiology Unit, St. Thomas Hospital, Kings College, London, UK; Dermatology Department, Hemel Hempstead General Hospital, West Herts NHS Trust, Herts, UK

## Abstract

There are many observations regarding the behaviour of melanoma which points away from sunshine as the main cause of this tumour. Incidence data shows that the increase is mostly seen for thin melanomas which cannot be attributed to sun exposure but increasing screening over the last 20 years. Melanoma behaves in a similar fashion all over the world regarding age of onset, gender differences and histological subtypes. An excess of naevi is the strongest risk factor for melanoma and their appearance and involution throughout life, and the differences in naevus distribution according to gender is giving us a lot of clues about melanoma biology. Melanoma like all cancers is a complex disease with the involvement of many common and low penetrance genes many of them involved in pigmentation and naevogenesis but these only explain a very small portion of melanoma susceptibility. Genes involved in melanocyte differentiation early on in embryogenesis are also becoming relevant for melanoma initiation and progression. Reduced senescence and longevity as well as body weight and energy expenditure are also relevant for melanoma susceptibility. These observations with links between melanoma and non-sun related phenotypes as well as gene discoveries should help to assess the relative contribution of genetic and environmental factors in its causation.

This review, whilst not wishing to dismiss sunshine altogether is attempting to redress the balance especially as the primary prevention of melanoma with avoidance of sun exposure has not been proven to reduce melanoma incidence and mortality and may be harmful.

## Melanoma incidence and mortality

The main argument for implicating sunshine as the cause of melanoma is the difference in melanoma incidence between Australia and Europe. The other argument is the rapid rise in melanoma incidence over the last 30 years which has been attributed to changes in sun seeking behaviour over the same period. [1,2]. However, there are some clues in the epidemiological data which, when looked at carefully, do not point to sunshine as being the main cause for these observations. When examining the incidence of melanoma in Australia, for example, the rapid rise in incidence has mainly been for very thin melanomas which have hardly had any impact on mortality which has remained fairly flat over the same period [2,3]. The delivery of dermatology services in Australia is very different from many European countries and especially the UK. Dermatologists and general practitioners in Australia offer rapid access melanoma screening following frequent public health campaigns and many naevi are excised during the screening process. These screening campaigns have led to a rapid rise in the number of very thin and borderline melanomas which in turn inflates overall incidence [4].

Comparing mortality and incidence data in melanoma is also important as it gives further clues to the types of melanomas removed following screening campaigns. The incidence of melanoma has risen dramatically in all countries where access to dermatologists is relatively easy and public health campaigns have been quite active. However, mortality has remained relatively stable and in some countries is even dropping with a widening between the mortality and incidence curves over the last 20 years [1].

This also supports the fact that most melanomas removed from screening campaigns are very thin and are likely to be biologically different. Data from Eastern European countries also supports this. A rise in incidence of melanoma in Eastern European countries has only occurred in countries where access to dermatologists has become easier with public health campaigns over that least 15 years. In other Eastern European countries, where this was not in place over the same period, the lower incidence and greater thickness of melanomas is comparable to figures seen in the UK 30 years ago [5]. Whilst there is no suggestion that we should not be detecting melanoma early, the impact of active screening and public health campaigns on melanoma incidence needs to be recognised and changes in sun exposure should not be blamed as the cause for this rapid rise in incidence without supporting evidence. The impact of screening on breast and prostate cancer incidence with the detection of many early lesions has been well documented and this is no different for melanoma [4].

## Melanoma behaviour: site, histological subtypes and gender

Another argument to downplay the role of sunshine in melanoma is the fact that melanoma is a tumour which behaves in a very similar fashion within similar ethnic groups all over the world despite very different levels of UV exposure. Population based melanomas outside the familial setting have a mean age of onset in the mid-fifties and this is constant in the UK or Australia [6]. If sunshine was such a driver for melanoma in Australia, this should, in theory, lower the age of onset. The relative proportions of different histological subtypes of melanoma such as superficial spreading, nodular melanoma and lentigo maligna (with invasion) are also very similar across all countries. The distribution of melanomas on the body is also telling. Melanomas in males tend to occur more commonly on the trunk whilst melanomas in females are mostly seen on the legs [7]. This observation is again somehow attributed to differences in sun exposure habits between males and females but the data does not support this as the difference in body sites according to gender is constant across all Caucasian populations irrespective of sun exposure. This difference is most likely to be explained by differences in melanocyte differentiation between males and females. Indeed when counting naevi in small children and following them up with age, boys and girls already differ in their naevus distribution with more naevi on the limbs especially arms and legs in girls and more naevi on the trunk in boys which reflects the distribution of melanomas in adults [8,9]. It is possible that intense and repeated sunlight exposure in childhood may increase the number of small naevi as total naevus counts are higher in Australia compared to the UK but it is likely that small lentigines are also miscounted as naevi on sun exposed sites [6]. Males also show consistently higher number of naevi than females and this is also seen in different parts of the world irrespective of sun exposure [8,9]. The types of naevi also vary greatly according to body sites as intradermal naevi which are mature lesions with a low risk of transformation into melanoma are common on the face for example but are more rarely seen on the limbs for examples. On the other hand, junctional and atypical naevi which are more unstable lesions in terms of melanoma risk are very rare on the face and are most common on the trunk and proximal limbs. Sun exposure again cannot explain these observations as the face is a chronically sun exposed site yet does not tend to have unstable melanocytic lesions such as junctional and atypical naevi. Specific gene expression in melanocytes from various body sites is likely to explain these observations and the mouse model provides clues about differences in behaviour of melanocytes on the cephalic region compared to limbs and dorsal areas and this has also been observed for fibroblasts [10].

There is also a significant increase in survival from melanoma in all populations in females so gender specific factors are crucial in the field of melanoma [1]. Brain tumours also show a significant increased survival in females and as melanocyte and neurones both come from the neural crest, it is likely that gender differences in neural crest cells differentiation explain these findings [11]. With the advances in genetics, these differences in naevi and melanoma behaviour according to histological subtypes and gender can hopefully be investigated in more detail but will need large and well phenotyped datasets to achieve this.

## Biology of naevi as strongest risk factor for melanoma

**Fig. 1:**
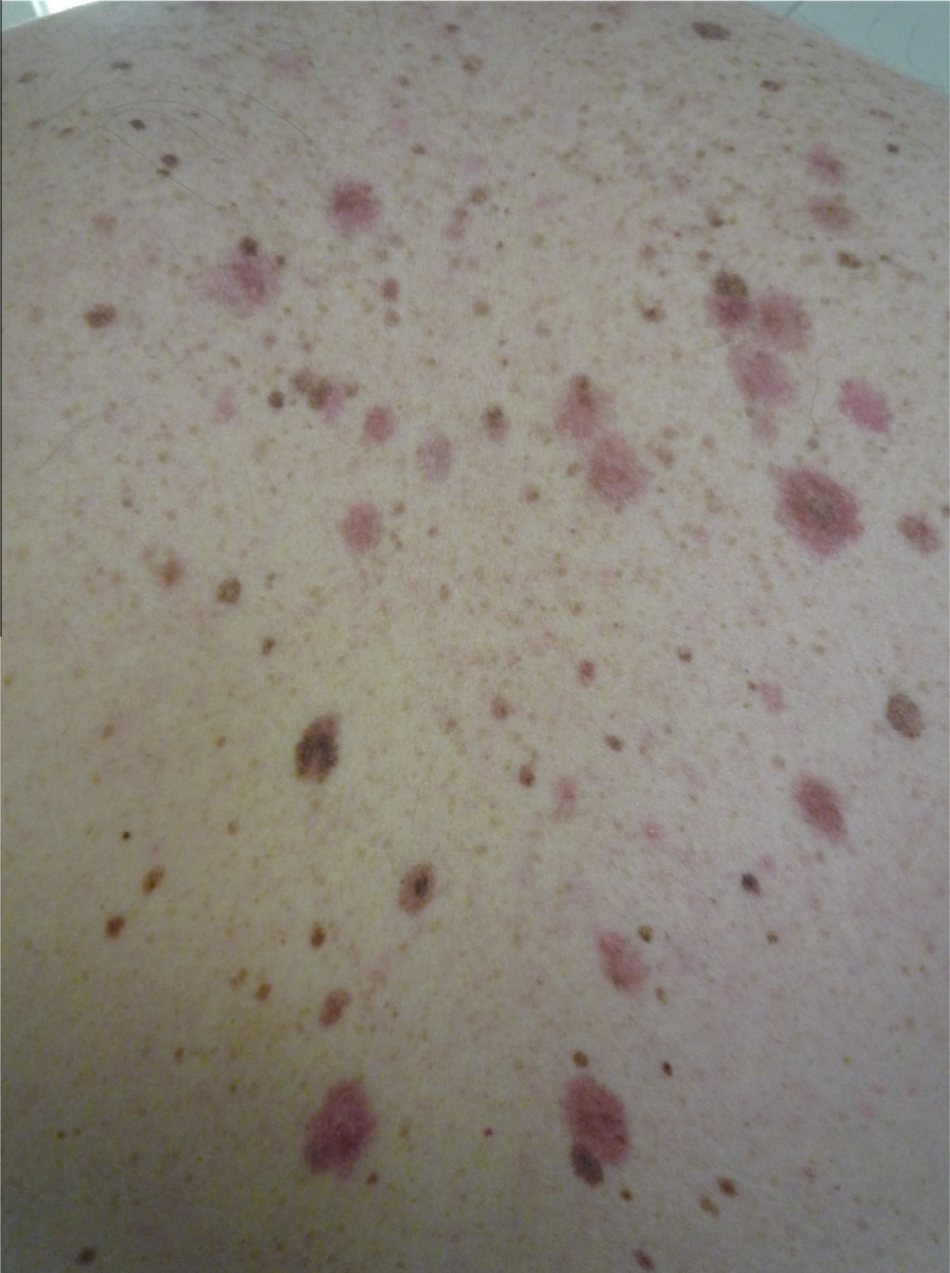
The atypical mole syndrome (AMS). Naevi genetics has helped in discovering new melanoma genes but this phenotype is also helpful as it does unravel some associations between melanoma risk factors and other phenotypes such as reduced ageing and longer telomeres.

The number and types of naevi (such as atypical naevi) is the strongest risk factor for melanoma in all Caucasian populations and the magnitude of the odds ratios (5–20) is much greater than any odds ratios ever reported for sun exposure and skin colour (1.5–2) (Fig. 1) [12,13]. Naevi confer the same magnitude of risk for melanoma at all latitudes showing again that sunlight is not that important for this association [14]. Looking into the biology of naevi is likely to lead to very interesting clues regarding the pathogenesis of melanoma. Melanocytes derive from the neural crest cells and their differentiation and migration to the skin occur during embryogenesis. This involves many genes such as MITF, WNT signalling, SOX10 and SOX 9 to mention only a few [15]. Interestingly, one of these genes, MITF has recently been reported to be involved later on in adulthood in melanoma susceptibility and invasion [16,17]. BRAF somatic mutations are also very common in naevi and melanoma and this led recently to the first therapy ever conferring an increased survival in melanoma [18,19]. Bastian and colleagues have looked at genetic signatures in melanoma tumours according to histological subtypes and body sites [20]. BRAF mutated melanomas are more common on the trunk and limbs compared to head and neck tumours and this again was thought to be due to differences in sun exposure but this has not been proven as yet. The association between anatomical site of melanoma and specific genetic signatures is quite likely to be explained by differences in melanocyte differentiation according to body sites programmed early on during embryogenesis as discussed earlier [10,21]. Naevi disappear from middle age onwards and this senescence is thought to involve oncogenes such as RAS and BRAF as well as tumour suppressor genes such as p16 or CDKN2A and possibly the telomere unit but the mechanisms of melanocyte senescence with age are poorly understood [22]. It is has, however, been reported recently that benign naevi do not appear to be permanently growth arrested via oncogene driven senescence as markers of senescence in naevi do not differ from early melanomas so what stops them from progressing into melanoma is still obscure [23]. Developmental and cancer genes appear very important in naevi formation and in turn melanoma susceptibility and hopefully this area of research will shed more light on stem cells genetics and melanoma risk.

When looking at melanoma families, CDKN2A/p16 germline mutations are found in roughly 25–40% of all melanoma worldwide [24]. Germline mutations in CDKN2A have also been linked to familial pancreas cancer yet this cancer has never been linked to sun exposure [25]. Melanoma and pancreas cancer can also cluster in the same families so these two tumours share genetic pathways [26]. For population based melanoma, recent large scale genome wide association studies in melanoma and naevi have discovered new common low penetrance genes (MTAP, PLA2G6, TERT and IRF4 for example) associated with both naevi number and melanoma [27–30]. MTAP and IRF4 have been implicated in cancer and immune responses respectively and IRF4 is also important in pigmentation [19,27]. MTAP is located closely to the CDKN2A locus and melanoma tumours often show loss of both loci and it is therefore possible that there are interactions between these two genes [27]. More recently, rare SNPs variants in MITF have been reported in melanoma and appear to act via naevi [16,17]. There are, however, many other genes involved in naevogenesis to be discovered. Interestingly variants in PLA2G6 associated with both melanoma and naevi in recent GWAS, have very recently been linked to Parkinson’s disease and the incidence of melanoma is greater in Parkinson cases than in controls [31,32]. This again generates some interesting questions regarding possible common genetic alterations in cells which share the same origin in the neural crest and sunshine is not an apparent link factor for these observations.

## Lack of significant photoageing in melanoma

**Fig. 2:**
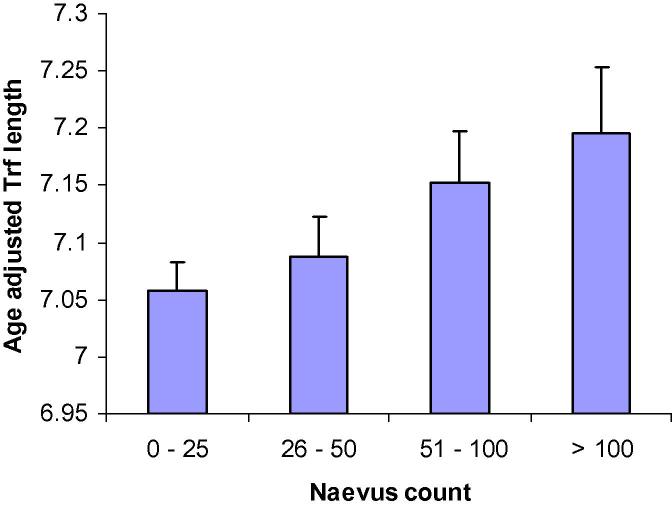
Naevus count categories in relation to telomere length. Bataille et al. 2007.

A further clue pointing away from sunshine as the main cause of melanoma is the lack of photoageing in melanoma. As sunshine is thought to be the main cause of melanoma one would expect that the deleterious effects of UV light would be visible with significant photoageing in melanoma patients. In fact, it is often the reverse as melanoma patients often have less photoageing with a lower prevalence of solar keratoses and solar lentigines compared to subjects susceptible to squamous cell and basal cell carcinomas for example (at the exception of head and neck melanomas which occurs in older age groups). In fact naevi and solar keratoses are often mutually exclusive and this led to the concept of different pathways in melanoma [33,34]. The observation that melanoma patients with a large number of naevi are showing less sun damage than controls may be explained by the discovery that individuals with a large number of naevi have longer telomeres than controls [35]. Telomere length was significantly associated with increasing naevus counts (Fig. 2). This delayed senescence in melanocytes is likely to be reflected into fibroblasts and keratinocytes hence the observed reduced photoageing. This has now been supported by two more recent studies showing that individuals with melanoma also have longer telomeres and this appears to be conveyed by an excess of naevi [36,37]. The TERT locus has also been associated with melanoma in GWAS analyses which further support the role of the telomere unit and possibly longevity for this tumour [38]. Recently, a germline mutation in the promoter of the TERT gene has been shown to explain melanoma susceptibility in a very large melanoma kindred which was also implicated in sporadic melanomas [39,40]. Large number of naevi and increased risk of melanoma therefore appear to be associated with reduced senescence and this may have provided a small survival advantage in evolution. It is speculated that this delayed senescence is what makes these individuals with high naevus counts with the atypical mole syndrome (Fig. 1) more prone to cancer as melanoma is often found in the context of family cancer syndromes and the longevity may be offset by an increased risk of cancers [21,25].

## Skin pigmentation and the MSH/MC1R pathway

Fair skin subjects are more likely to get melanomas compared to darker skin types and this may be the strongest piece of evidence linking UV sensitivity with melanoma from epidemiology studies. However, the risk associated with sunburns and fair skin is small with risk factors in the order of 1.5–2 compared to relative risks of 5–30 for multiple atypical naevi without or with family of melanoma respectively [12,13]. Once adjusted for skin type, many sun exposure measures do no longer confer a risk for melanoma so it is not a fixed amount of sun exposure which matters but the host response. It is possible that fair skin subjects may have other, yet unknown, risk factors for melanoma rather than just being fair and sun sensitive. The MSH/melanocortin pathways linked to skin pigmentation is very complex and has important functions regarding immune and neuroendocrine responses as well [41]. Many pigmentation genes linked to melanoma in recent GWAS such as TPCN2, ASIP, KIT, NCKX5, TYR, IRF 4, OCA2 and TYRP1 have been linked to melanoma and some of them like MC1R have dual roles in pigmentation and immune responses but many other functions of these genes remain to be discovered [30]. The melanocortin system containing endogenous agonists (POMC derived peptides) and agonists (agouti protein and AgRP (agouti-related peptide) as well as five MCR subtypes from (MC1R to MC5R) also regulate energy and glucose homeostasis and melanoma has been linked to increased BMI [42–44]. This association between BMI and melanocortins and, in turn, pigmentation, was clearly demonstrated in the mouse model before [42]. Furthermore, GWAS studies in obesity based on more than 200,000 individuals have confirmed that genes in this pathway such as POMC and MC4R are associated with obesity [45]. More recently, melanoma susceptibility has been linked to the FTO gene which is also an important gene for BMI [46].

## Vitamin D

An important and neglected area of research until very recently is the role Vitamin D in the susceptibility and survival of melanoma. Vitamin D is associated with sun exposure, pigmentation, BMI and telomere length so it is linking many risk factors for melanoma discussed above [47–49]. Newton Bishop advanced the field recently showing that low vitamin D levels are common in melanoma patients and have a deleterious impact on risk and survival [50]. It is therefore important to dissect these associations further not only to produce some safe recommendations regarding sun exposure but also to understand the complex relationships between various melanoma risk factors.

## Recent evidence of sun exposure in melanoma

It may be argued that this review did not debate enough the sun exposure evidence in melanoma. The link between melanoma and UV radiation has been based on many epidemiological studies, migration studies, animal models and cell biology over the last 30 years and it is not the scope of this review to go through all this evidence and assess the relevance of these observations for human melanomas. This would take too long and should be part of another manuscript. This review’s aim is to redress the balance and present evidence of important non sun related melanoma risk factors which are often ignored in the literature. In terms of recent evidence, genome sequencing of melanoma tumours have shown that the number of all types of genetic alterations exceed 70,000 and that many of the point mutations are C-T pyrimidine dimers which in principle supports sun exposure in melanoma [51]. C-T mutations are, however, also found in genome sequencing of other tumours which have no link to sun exposure [52]. There is also the possibility that the new methylome studies in melanoma will show some effect of sun exposure in turning genes on and off which may affect melanoma risk. However, it is too early at this point as the very recent methylome studies on human melanoma have not been correlated with environmental exposure as it is relatively difficult to have accurate sun exposure data in these studies [53].

In summary, recent advances in genetics with good collection of environmental data will hopefully help to dissect the relative contribution of environmental and genetic factors in the causation of melanoma. The arguments used to support sunshine as the main cause of melanoma do not always stack up. This review does not wish to dismiss sunshine but rather present another view on melanoma biology. It is clear that sunshine causes significant photoageing and increase skin cancer risk with a significant burden to health services so excessive sun exposure is not advocated. But it should be recognised that drastically reducing sun exposure in Caucasians may have deleterious effects which may take many years to unravel and has not been successful in reducing melanoma incidence. Sunlight is unlikely to be blamed for the rapid rise in melanoma incidence seen over the last 30 years and instead changes in screening for melanoma are probably responsible for a rapid rise in early melanomas. New common low penetrance genes discovered via genome wide association studies and melanocyte biology show that melanocyte differentiation is very complex and is linked to other phenotypes such as longevity, BMI and energy expenditure amongst many others. Gender is also a very important modifying factor in naevi and melanoma biology and gender effects on naevi and melanoma needs to be explored further. More genome data as well as methylome studies are eagerly awaited to further elucidate the driver mutations and epigenetic changes in melanoma. However, the “at risk phenotype” should not be ignored and studies should not focus on melanoma tumours only. Vitamin D is linked to so many melanoma risk factors and appears to be protective for melanoma so more research is needed especially as Vitamin D deficiency is now becoming very prevalent in all Caucasian populations.

## Conflict of interest

None.
